# Enhanced Precision in Genioplasty: A Novel Intraoperative Spatial Repositioning Using Computer-Aided Design and Manufacturing Technology and a Holographic Mixed Reality Application

**DOI:** 10.3390/jcm12237408

**Published:** 2023-11-29

**Authors:** Masahide Koyachi, Keisuke Sugahara, Kotaro Tachizawa, Akihiro Nishiyama, Kento Odaka, Satoru Matsunaga, Maki Sugimoto, Chie Tachiki, Yasushi Nishii, Akira Katakura

**Affiliations:** 1 Department of Oral Pathobiological Science and Surgery, Tokyo Dental College, 2-9-18 Kandamisaki-cho, Chiyoda-ku, Tokyo 101-0061, Japan; koyachim@tdc.ac.jp (M.K.); tachizawakotaro@tdc.ac.jp (K.T.); nishiaki@tdc.ac.jp (A.N.); sgmt@med.teikyo-u.ac.jp (M.S.); katakura@tdc.ac.jp (A.K.); 2 Department of Oral and Maxillofacial Radiology, Tokyo Dental College, 2-9-18 Kandamisaki-cho, Chiyoda-ku, Tokyo 101-0061, Japan; odakakento@tdc.ac.jp; 3 Department of Anatomy, Tokyo Dental College, 2-9-18 Kandamisaki-cho, Chiyoda-ku, Tokyo 101-0061, Japan; matsuna@tdc.ac.jp; 4 Innovation Lab, Teikyo University Okinaga Research Institute, 2-16-1 Hirakawacho Chiyoda-ku, Tokyo 102-0093, Japan; 5 Department of Orthodontics, Tokyo Dental College, 2-9-18 Kandamisaki-cho, Chiyoda-ku, Tokyo 101-0061, Japan; tachikichie@tdc.ac.jp (C.T.); nishii@tdc.ac.jp (Y.N.)

**Keywords:** computer-aided design and manufacturing, mixed reality, genioplasty, orthognathic surgery

## Abstract

Genioplasty is performed for the orthognathic surgical correction of dentofacial deformities. This article reports a safe and accurate method for genioplasty combining a novel three-dimensional (3D) device with mixed reality (MR)-assisted surgery using a registration marker and a head-mounted display. Four types of devices were designed based on the virtual operation: a surgical splint with a connector; an osteotomy device; a repositioning device; and a registration marker. Microsoft HoloLens 2 and Holoeyes MD were used to project holograms created using computed tomography (CT) data onto the surgical field to improve the accuracy of the computer-aided designed and manufactured (CAD/CAM) surgical guides. After making an incision on the oral vestibule, the splint was fitted on the teeth and the osteotomy device was mounted at the junction site, placed directly on the exposed mandible bone surface. Temporary screws were fixed into the screw hole. An ultrasonic cutting instrument was used for the osteotomy. After separating the bone, a repositioning device was connected to the splint junction and bone segment, and repositioning was performed. At the time of repositioning, the registration marker was connected to the splint junction, and mandible repositioning was confirmed three-dimensionally through HoloLens 2 into the position specified in the virtual surgery. The rate of overlay error between the preoperative virtual operation and one-month postoperative CT data within 2 mm was 100%. CAD/CAM combined with MR enabled accurate genioplasty.

## 1. Introduction

Genioplasty is a type of orthognathic surgery that can improve functionality and cosmetics by adjusting the shape of the mental region in three dimensions. Hofer et al. applied this method to cadavers for the first time, and Gillies and Millard applied it to patients using an extraoral approach [1]. Trauner operated on a patient using an intraoral approach, which they named “genioplasty” [2]. The osteotomy line and the movement of the segment impact the surgical outcomes. Genioplasty is traditionally performed based on the surgeon’s experience, skills, and intraoperative assessment.

In recent years, surgery utilizing digital technology and equipment has developed and is rapidly gaining popularity in the field of maxillofacial surgery [3,4]. Developments in three-dimensional (3D) technology over the past decade have popularized the use of 3D printers, especially in orthognathic surgeries. The creation of a surgical guide using computer-aided design/computer-aided manufacturing (CAD/CAM) technology is linked to increased surgical accuracy, improved safety, and a reduction in operating times [5]. There are many reports on the use of 3D devices to support genioplasty [6]. The mainstream method combines a cutting guide and a positioning guide that accurately reproduce 3D movements. However, confirming the movement of the bone segment during the operation, as planned preoperatively, is difficult. On the other hand, mixed reality (MR) is being applied for performing surgery while projecting preoperative information onto the surgical field. The use of MR technology also improves the safety and accuracy of surgery and its usefulness in tooth extraction has been reported in oral and maxillofacial surgeries [7]. Furthermore, the accuracy of surgery can be improved by combining CAD/CAM and MR [8,9]. However, there have been few reports on its application to genioplasty. Therefore, this clinical report aimed to report a novel MR method for genioplasty and verify its accuracy.

## 2. Materials and Methods

### 2.1. Ethical Statement

The study design was approved by the Ethics Committee of Tokyo Dental College, Tokyo, Japan (No. 794, 1054). Written informed consent was obtained from the patient for publication.

### 2.2. Procedure

**Figure 1:**
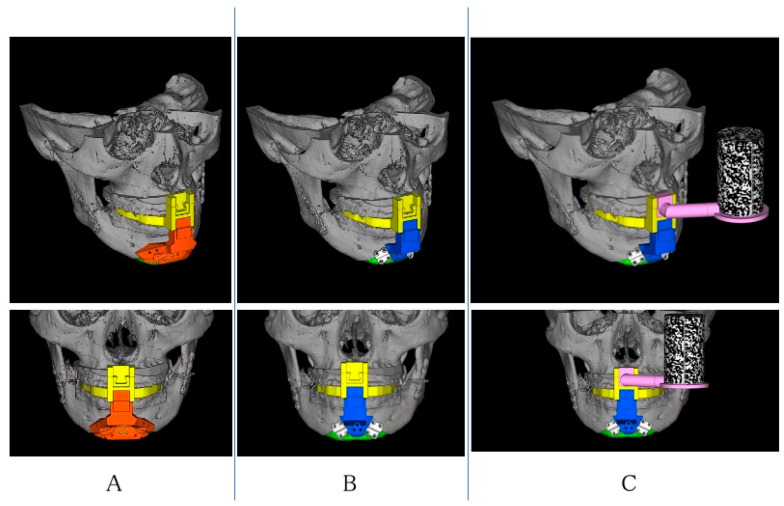
Computer-aided design of the three-dimensional devices: (**A**) splint (yellow) and osteotomy device (red); (**B**) repositioning device (blue); and (**C**) registration marker (pink and monochrome mosaic).

**Figure 2:**
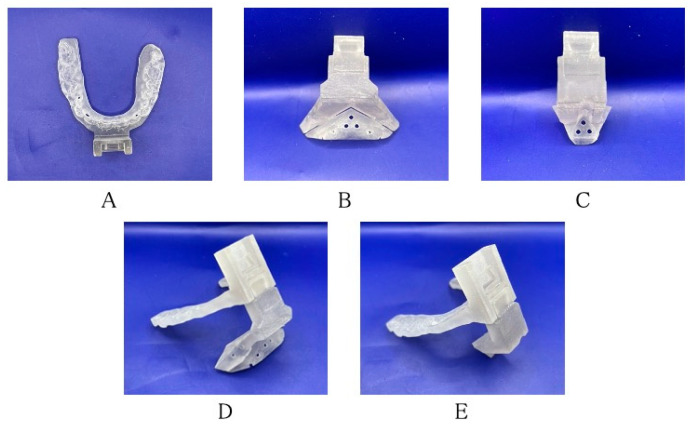
Three-dimensional devices made by a three-dimensional printer: (**A**) surgical splint; (**B**) osteotomy device; (**C**) repositioning device; (**D**) surgical splint connected to the osteotomy device; and (**E**) surgical splint connected to the repositioning device.

Three-dimensional images of the mandible were reconstructed using computed tomography scans with a 0.6-mm slice thickness (CT; Somatom Definition AS, Munich, Germany). The CT data were converted to anonymized Digital Imaging and Communications in Medicine (DICOM) data. Since orthodontic brackets and prosthetic artifacts make it difficult to reproduce the dentition accurately, optical impressions of the occlusal surface morphology were taken (Trios; 3Shape, Copenhagen, Denmark). In addition, the plates were converted to standard triangulated language (STL) data. Based on the STL data of the mandible reconstructed from the CT Digital Imaging and Communications in Medicine data and digital impressions, segmentation was performed using Mimics (Materialise, Leuven, Belgium), surgical simulation was performed using the simulation software ProPlan CMF^™^ (Materialise), and CAD software (Magics; Materialise) was used for virtual genioplasty. Figure 1 shows the devices used in this report. For these CAD designs, the technique established for Le Fort I osteotomy was applied [8]. In addition, the shape and size of the registration marker, which had room for improvement, was modified. The size of the registration marker was made smaller than before so as not to interfere with the operative field. The shape of the registration marker was cylindrical (with a diameter of 33 mm and a length of 56 mm), so that the hologram could follow it from any side. Four types of devices were designed based on the virtual operation: a surgical splint with connectors; an osteotomy device (Figure 1A); a repositioning device (Figure 1B); and a registration marker (Figure 1C). These devices were fabricated with a polyjet 3D printer (Objet 260 connex; Stratasys, Eden Prairie, MN) using biocompatible resin (MED 610; Stratasys). The splint was designed by pulling out a simple shape from the region, including the interproximal space below the orthodontic brackets of the anterior teeth (Figure 2A). The osteotomy and repositioning devices were developed in such a way that they could be attached to the splint and switched in or out at the lower junction site, and so that a registration marker could be attached to the upper junction.

The osteotomy device had a slit for the ultrasonic cutting instrument that reflected the osteotomy line. In the center, where the osteotomy device was in contact with the mandibular surface, three drilling guides were placed for the 2 mm diameter repositioning screws, which were the same diameter as the temporary fixing screws. Four drilling guides for the screws were designed for repositioning on both sides of the osteotomy line. These holes were positioned to reflect the plating position assumed during preoperative simulation (Figure 2B). At the center of the repositioning device, in the surface that matched the shape of the mandible after movement, three 2 mm diameter holes were designed to fit the repositioning screws (Figure 2C). A dovetail joint was used to connect the splint, osteotomy device, repositioning device, and registration marker (Figure 2D,E). There was space between the upper part of the osteotomy and the repositioning device; hence, they did not interfere with the inverted flap. The connector was designed by checking the soft tissue data in advance to avoid interference with the lips.

#### 2.2.1. MR Application

The STL data were converted from the virtual simulation into a hologram application. The application was created online by uploading STL data to the Holoeyes MD (Holoeyes, Tokyo, Japan) application. Data were then installed on a head-mounted display (HMD) (HoloLens 2; Microsoft, Redmond, WA). The hologram was projected onto the surgical field using HoloLens 2. When HoloLens 2 recognizes the registration marker, the hologram that reflects the preoperative virtual operation plan is superimposed on the operative field. This allows the surgeon to confirm that the mandible has been repositioned during surgery as planned. The hologram can change in size, angle, and transparency; switch freely to the segment to be displayed; and display any cross-section by finger gestures.

#### 2.2.2. Surgical Procedure

An incision was made anteriorly in the labial vestibule. The splint was placed between the maxillary and mandibular dentition. The osteotomy device was then placed directly on the exposed bone surface and fixed using screws. When inserted, the splint and osteotomy device was fitted on the bone surface of the mental region teeth without interfering with the gingival flap. Subsequently, the osteotomy device was guided into the bone surface on the mandible through the dovetail joint of the connector and then fixed rigidly to the bone. 

**Figure 3:**
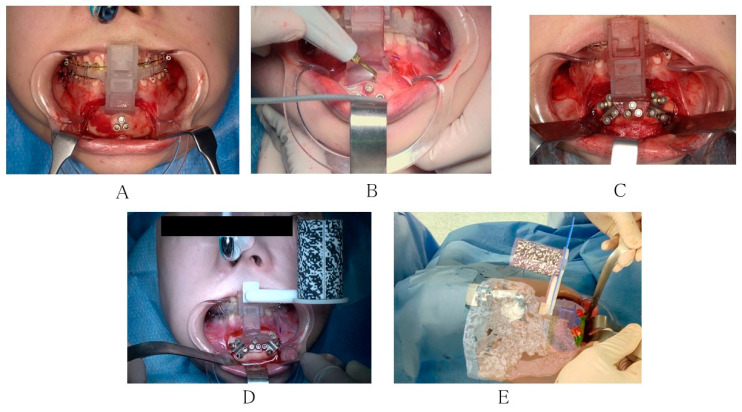
Intraoperative photo: (**A**) using the osteotomy device; (**B**) osteotomy using an ultrasonic cutting instrument; (**C**) repositioning device and prefixed plates attached; (**D**) repositioning device and registration marker connected to the splint; and (**E**) projecting the hologram through HoloLens 2.

The osteotomy was performed using an ultrasonic cutting instrument along the slit of the osteotomy device (Figure 3A,B). The repositioning device was fixed instead of the osteotomy device using the previously drilled holes as the reference points. Following that, the bone segment was fixed to the repositioning device. The bone segments were guided to the planned position using the repositioning device (Figure 3C). After connecting the registration marker to the splint junction, the correct repositioning of the mandible was confirmed in 3D using HoloLens 2. Once it was confirmed to be in the position specified in the virtual surgery, the repositioning device was removed, and two resorbable four-hole plates were used to fix the chin segment to the main body of the mandible (Figure 3D,E).

### 2.3. Evaluation

Postoperative evaluation was performed using Mimics and Magics to compare preoperative virtual operation 3D images (Tv) with CT images (T1) taken one month postoperatively. Superimposition of preoperative and postoperative image data and evaluation was performed by GOM Inspect (GOM, Braunschweig, Germany). As the body of the mandible is not affected by genioplasty, it was used as the basis for image superimposition. The initial superposition was performed at three arbitrary points, and then the main superposition was performed by the partial-best-fit method [10].

To compare only the bone surfaces, the resorbable plates, screws, and artifacts were removed from postoperative CT images. The surface of the bone segment was selected as the region of interest for evaluation, and the osteotomy surface of the bone segment and the screw holes used for temporary fixation were excluded from the region of interest. Surface deviation was evaluated using superimposed 3D images [11]. The errors between the Tv and T1 bone surfaces were measured. Based on previous reports, threshold values of <2 mm and 1 mm were set for Tv and T1, respectively, and the percentage of errors below these values was calculated [12]. Additionally, the maximum and minimum error values and the average error value were calculated.

## 3. Results

In the first month following the surgery, there was no sensory abnormality of the mental nerve or damage to the root of the tooth. Furthermore, no detachment or fracture of the fixing screws and plates were observed, and no other abnormal findings could be identified after surgery. The standard deviation of registration errors was 0.19 mm. The percentage of bone measurement error within 2 mm between the Tv and T1 images was 100%, and within 1 mm was 90.9%, respectively. The maximum value of the error comparing Tv and T1 was 1.825 mm, the minimum value was 0 mm, and the average error value was 0.067 mm.

## 4. Discussion

The shape of the chin strongly influences the visual impression of a patient’s face [13,14]. In particular, chin deformity due to chin overgrowth or undergrowth can cause lip dysfunction and esthetic disturbances in patients. Therefore, it is important to perform virtual surgeries before surgery and accurately reflect them during surgery. In recent years, in the field of oral and maxillofacial surgery, techniques for creating full-scale 3D models of maxillofacial bones and surgical guides based on image data from CT and magnetic resonance imaging have become widespread, and are being used in various surgeries [15,16]. In orthognathic surgery, surgical support devices made with a 3D printer using biocompatible resin and a patient-specific implant using a 3D metal printer are used to improve the accuracy of surgery [17]. In this method, we designed a connecting part in a single surgical splint, and it was possible to switch between an osteotomy device and a repositioning device at the bottom connecting part. Thus, it is hoped that costs related to device manufacturing can be reduced. Furthermore, a marker for superimposing the hologram and the surgical field can be connected to the upper connecting part of the surgical splint. This is a novel device that differs from genioplasty devices reported so far.

Using CAD/CAM and a holographic MR application for genioplasty, the rate of overlay error between preoperative virtual operation and postoperative CT data within 2 mm was 100%, and within 1 mm was 90.9%. In previous studies, the average error reported was 0.06 to 0.70 (maximum 2.94 mm, minimum 0.00 mm) [12,18], suggesting that surgery was performed with high precision in the present case. In this report, the combination of a device made with a 3D printer and MR contributed to high-precision surgery. By superimposing the virtual operation hologram on the operative field during surgery, it was possible to confirm whether repositioning of the chin was performed accurately in 3D, and whether the plate was positioned as planned. Conventionally, methods for confirming the movement of the jawbone during surgery using a navigation system have been reported [11]. However, the method using a navigation system requires a large and expensive device and needs to be calibrated before surgery and re-calibrated if it falls out of alignment during surgery. MR-equipped HMDs are now being used for navigation in various surgeries [19,20]. A surgeon wearing an HMD can view a 3D hologram created from clinical image data and perform surgery without taking their eyes off the patient. HMDs, such as the Microsoft HoloLens, are easy to carry and can be intuitively operated with gestures and voice. In addition, the updated HoloLens 2 provides higher resolution and more intuitive operation [21]. In this method with the HoloLens 2, the surgeon starts the operation while wearing the HMD, and it takes less than a few seconds to superimpose the hologram and the surgical field. In addition, this device is easy to handle because it is thin and does not interfere with the operative field. Even when surgeons move their lines of sight away from the surgical field, the hologram is fixed to the spatial coordinate system; thus, there is no need for recalibration. Safe and accurate surgery is possible when using the HMD with an ultrasonic cutting device, and there is a low risk of damage to the tooth roots and nerves during surgery. The most important aspect of this technique for accurate movement of the bone fragments is the fitting of the splint to the dentition; however, patients undergoing genioplasty often have orthodontic brackets and metal artifacts on CT images. Therefore, it is difficult to determine the tooth shape. Designing 3D devices can also be challenging when there are prostheses such as crowns, bridges, implants, metal posts, and cores on teeth. In such cases, optical impressions can be used to create STL data for an appropriate dentition morphology. In this method, CT and optical impression data are combined using Mimics and Magics to create an optimal 3D device. This method has the advantage of being able to confirm, in 3D, whether the chin is moving according to the virtual operation during surgery. A limitation of this method is that most of the surgical accuracy depends on the 3D device. As MR has already been used in surgery in other fields, it will be possible to perform orthognathic surgery with only MR surgery support, without using 3D devices, in the near future [22]. However, because the technology has not progressed to that extent, the method of combining CAD/CAM and MR is effective.

## 5. Conclusions

Utilizing augmented holographic simulations over the surgical field enabled the confirmation of spatial repositioning of the mandible and the precise placement of planned plates. This method enhanced the accuracy in relocating bone fragments. Thus, the combined use of CAD/CAM and a holographic MR application can allow for intraoperative spatial verification of mandibular movements as per virtual manipulations. This integration can serve as a surgical guide, augmenting the precision of the technique and contributing significantly to high-accuracy surgical procedures.

## Data Availability

Data are contained within the article.
